# Hematology and Blood Chemistry Reference Values of Captive Adult Black-Faced Ibis (*Theristicus melanopis melanopis*)

**DOI:** 10.3390/ani10122227

**Published:** 2020-11-27

**Authors:** Alonso Silva, Paola Mujica, Evelyn Valdés, Hernan Cañon-Jones

**Affiliations:** 1 Parque Safari Chile, Ruta H-30, Km 5, Camino a Doñihue S/N, Rancagua 2840169, Chile; alonsosh.mv@gmail.com; 2 Núcleo de Investigación Aplicada en Ciencias Veterinarias y Agronómicas, Universidad de Las Americas, Santiago 7500972, Chile; pmujica@udla.cl (P.M.); evelyn.valdes@udla.cl (E.V.)

**Keywords:** conservation health, rehabilitation, ecosystems, wild bird health, wild bird welfare

## Abstract

**Simple Summary:**

Wildlife and human interactions are increasing all over the world, with many injured wild animals needing to be treated or rehabilitated. Reference values are of great help when treating injured animals. The black-faced ibis is a bird common in South America and is one of the few ibises in this region. The human population has increased the risk of interaction with these birds, and rehabilitation centers are receiving injured birds. The aim of this work was to establish the normal or reference hematology and blood chemistry values in this species. The result may be used to elucidate the health and welfare status of injured or recovering black-faced ibis in rehabilitation centers and to act with the required therapeutic measures. This in turn may increase rates of survival and may indicate the correct moment of returning of the birds back to the wild while contributing to the conservation of this species.

**Abstract:**

Hematology and blood chemistry reference values in wildlife animals are considered a key element to evaluate their health and welfare status. The incidence of birds rescued is increasing, thus, rehabilitation centers worldwide need valid reference values to improve medical care for wild individuals. The objective of this study was to obtain the reference values of the adult black-faced ibis (*Theristicus melanopis*). Blood was taken from adult rehabilitated birds and analyzed to obtain red and white line values such as hematocrit, hemoglobin, mean corpuscular volume (MCV), mean corpuscular hemoglobin (MCH), mean corpuscular hemoglobin concentration (MCHC), heterophils, lymphocytes, monocytes, eosinophils and basophils. Values for blood glucose, proteins, albumin, globulin, calcium, phosphorus, blood urea nitrogen (BUN), creatinine, alkaline phosphatase, aspartate aminotransferase (AST), gamma-glutamyl transpeptidase (GGT), lactate dehydrogenase (LDH), creatine phosphokinase (CPK) and albumin:globulin (A/G) ratio were also obtained. The results were similar to others reported for species of the Threskiornithidae family (bald, glossy and Puna ibises), but showed higher values for white blood cells (WBC), heterophils, monocytes and heterophil-lymphocyte (H/L) ratio, but lower values for basophils and eosinophils. Moreover, higher values in albumin, BUN and CPK were observed. This is the first report of the hematology references values for the black-faced ibis showing differences to other closely related species. The results may be of use in rescue and rehabilitation centers for animal welfare and health assessments of the black-faced ibis.

## 1. Introduction

The black-faced ibis (*Theristicus melanopis*) is a native bird of Chile and is also found in some areas of Peru and Argentina [1,2]. There are only a few studies of this bird, mostly on ecology [3,4] and reproduction biology [5] due to the limited captive population and the complicated capture methods in the wild. It is categorized as a least concern species in the International Union for Conservation of Nature Red List [2]. However, the current legislation in Chile [6] classified *T. melanopis* as beneficial to agriculture and considered it as an endangered species in the north, vulnerable in the central and southern regions and least concerned in the austral region.

The black-faced ibis can inhabit multiple landscapes such as dry grasslands, swampy valleys, river banks or agricultural fields [7], and its diets consist of insects, annelids, and amphibians [8]. However, much of their habitat has been reduced because urbanization has increased [9]. This phenomenon may have been the cause of the increment in the number of black-faced ibises received in wildlife rehabilitation centers, mainly due to traumatic injuries, becoming the most common species received for rehabilitation during 2017 in Chile (personal communication with the Wildlife Rehabilitation Center “Metrenco” and Rescue Centre for Wildlife “CEREFAS” of Universidad Austral, Chile).

In addition, available information has shown limited success in releases back into the wild from rehabilitation centers worldwide [10,11].

Hematological and blood chemistry variables for wild birds are scarce, and those published have proved to be good indicators of the health and welfare status of wild birds in rehabilitation centers [12]. For example, in a rehabilitation centers, a low packed cell volume (<50%) was related to high mortality risk in raptors [13], with similar findings for adult African penguins (*Spheniscus demersus*) [14]. These studies showed the importance of having reference values in rehabilitation centers for birds at the national or regional level, thus ensuring a correct determination of health and welfare.

There are no studies considering the range for hematology and blood chemistry variables in the blacked-faced ibis. This lack of information may deter veterinarians from performing proper evaluations of health and welfare status of this specie. Furthermore, due to the wide geographical range of this species, it may be of importance to have normal range values specifically for the black-faced ibis populations in Chile. This is sustained from the evidence reported in other species of wild bird such as raptors, psitaccids and other ibises, showing that hematological variables can differ from geographically different regions attributed mainly to diet [15,16,17].

This study was carried out to establish the normal hematological and blood chemistry values in the black-faced ibis, and differences related to sex and sampling location were also assessed. This information could be used as a complementary tool for veterinary medicine in rehabilitation centers.

## 2. Materials and Methods

The samples were collected from clinically normal adult black-faced ibises that were born and held in captivity. The study was conducted in accordance with national legislation on the use of animals for research and ARRIVE guidelines [18]. A total of 22 birds were sampled across different rehabilitation centers in Chile (La Serena, Santiago, Rancagua and Linares cities) from 29°54′16.31″ S to 35°50′48.01″ S. This territorial coverage represents the north, central and south-central zones of Chile, where the majority of the human population exists and bird interactions can occur. All birds were clinically normal with no sign of illness. The sex of birds was determined and recorded by observation of cloacal region during sampling. Birds were manually immobilized, and blood samples were taken from the brachial vein and placed in ethylenediaminetetraacetic acid (EDTA) tubes. Samples were immediately stored between 2 to 8 °C and submitted to the certified diagnostic laboratory (Laboratorio Veterinario Especializado, VetLab, Santiago, Chile) within 24 h of sampling.

Samples were analyzed by the private veterinary laboratory using an automatic hematological analyzer (BC-3600, Mindray^®^). Hematology profile was obtained for the 22 birds, and serum biochemistry profiles were obtained from 11 bird samples. The reduced sample size for blood biochemistry was due to small final volume obtained in the 11 samples. Hematology variables were red blood count (RBC), hematocrit, hemoglobin, mean corpuscular hemoglobin (MCH), mean corpuscular hemoglobin concentration (MCHC), mean corpuscular volume (MCV), white blood cells (WBC), heterophils, eosinophils, lymphocytes, monocytes and the heterophil/lymphocyte ratio (H/L ratio). Automatic blood chemistry was carried out using an analyzer (BS-480, Mindra^®^). Glucose, total proteins, albumin, globulin, albumin:globulin ratio (A/G), calcium, phosphorus, blood urea nitrogen (BUN), creatinine, alkaline phosphatase (ALP), aspartate aminotransferase (AST), gamma-glutamyl transpeptidase (GGT), lactate dehydrogenase (LDH) and creatine phosphokinase (CPK) were determined.

Normality tests were carried out using the Kolmogorov–Smirnov test. Means, standard deviations, minimum and maximum values and quartiles were calculated according to standard and suggested methods to obtain references values in animals [19]. Geographical differences in RBC, hemoglobin, MCH, MCHC, MCV, WBC, heterophils, eosinophils, basophils, lymphocytes and monocytes were detected using analysis of variance and for hematocrit, and for the H/L ratio, the Kruskal–Wallis test was used. To detect any correlation between continuous variables, a Pearson test was carried out. All statistical analyses were carried out using R software [20].

## 3. Results

The mean ± standard deviation, minimum and maximum values and quartiles for each hematological variable can be observed in Table 1.

Likewise, blood chemistry variables are shown in Table 2. All variables adjusted to normality except basophils, eosinophils, monocytes, H/L ratio, BUN and A/G ratio (*p* < 0.05).

There were no statistically significant differences in hematological (Table 3, *p* > 0.05) or biochemistry variables (Table 4, *p* > 0.05) in regard to sampling location.

Additionally, no statistical differences were found between the sex of birds for hematological variables (Table 5, *p* > 0.05) or blood biochemistry variables (Table 6, *p* > 0.05).

## 4. Discussion

The results presented in this communication are the first attempt to establish reference values for the black-faced ibis in Chile. The sample size in our study is small, but it is common for wild birds and is in accordance with guidelines for determination of reference values in wild animals [21]. However, the values for hematology were relatively similar to other stork species, such as the northern bald ibises (*Geronticus eremita*) [22], Puna ibis (*Plegadis ridgewayi*) [23] and white and black storks (*Ciconia ciconia* and *Ciconia nigra*) [24,25,26]. For example, our results showed higher values for WBC, heterophils, monocytes and H/L ratio, but lower values for basophils and eosinophils than those reported for bald ibises [15,27]. In contrast, no differences in hematology reference values of glossy ibises (*Plegadis falcinellus*) [28] or Puna ibises [23] were observed. A plausible explanation may be related to the habitats and dietary differences of the species, where bald ibises inhabit coastal areas of Africa [29], while the glossy and Puna ibises inhabit inland marshes and flatlands of Europe, North and also South America, similar to black-faced ibises [30,31]. Furthermore, different types of diet may impose dietary deficiencies, such as iron, which could have an effect on some blood components such as hematocrit or hemoglobin [32,33]. Moreover, dietary composition may have an impact on exposure to pathogens (virus, bacteria, protozoa, and coccidias) which may increase numbers of immune system cells such as lymphocytes and monocytes [34] and trigger metabolic changes that could be observed in blood chemistry variables, such as those related to liver function [35].

There are only a few studies exploring blood chemistry in the Threskiornithidae family, and the results from this study showed differences in some of these variables. The blood chemistry values were similar to the values reported for adult bald ibises [27], but we obtained higher values for phosphorus and LDH and lower values for calcium in the black-faced ibises analyzed in this study. In contrast, the sampled birds exhibited higher values in albumin, BUN and CPK when compared to Puna ibises [23]. There is no clear explanation for these differences, and they could be species-specific rather than from stress. Future studies should be conducted to elucidate plausible explanations for the observed differences in some blood chemistry parameters in ibises.

Stress, more specifically chronic stress, has been biological well characterized in many human an non-human animal species and may trigger a state of continuous catabolism affecting different systems that can be reflected in the modification of blood chemistry variables [36] or hematology, such as the H/L ratio [37].

Furthermore, no differences were observed for the sex of birds or sampling location, suggesting that both hematology and blood chemistry variables are not affected by these factors. More studies should be conducted to ensure a large sample size and confirm these preliminary results.

Finally, it is important to point out that, although the number of total animals sample are in accordance with or even higher than many other studies in wild birds [15,16,25,28] and recommendations for obtaining reference values in wild animals [19,21], it is recommended to increase the number of individuals to increase the statistical power and analysis. This will depend on the number of birds entering rehabilitation centers, and the sampling should be carried out before returning them to the wild.

## 5. Conclusions

This study provided the first report for hematological parameters and blood chemistry reference values in adult black-faced ibises in Chile. Most of the results obtained were similar to others previously published for other ibises’ species, but differences both in hematology and blood chemistry were observed. These differences may be specie-specific or a result of the different diet of the birds at a regional or national level, as there were no differences among various sampling locations of Chile. These preliminary results should be further explored in terms of number of sampled birds, age and seasons. However, these results may be of use in rescue and rehabilitation centers as a tool to make informed and scientifically based decisions for animal welfare and health in the black-faced ibis living in the southern American hemisphere.

## Figures and Tables

**Table 1:** Mean ± standard deviation, minimum and maximum values and quartiles and of hematological variables of captive black-faced ibis (*Theristicus melanopis*) (N = 22).

Variable	Mean +/− SD	Min	Max	2.5%	50%	97.5%
RBC (10^6^ cells/μL)	3.03 +/− 0.46	1.81	3.94	2.08	3.04	3.75
Hematocrit (%)	49.9 +/− 4.4	40.0	56.0	40.0	50.5	56.0
Hemoglobin (g/dL)	16.7 +/− 2.2	9.8	19.9	11.3	17.0	19.6
MCH (pg/cell)	57.2 +/− 4.0	50.6	67.4	51.3	56.5	65.1
MCHC (g/dL)	33.2 +/− 2.4	25.0	36.0	27.6	34.0	35.7
MCV (fL)	173.4 +/− 17.6	142.0	220.0	147.2	172.0	211.6
WBC (cell/μL)	9704 +/− 4249	3800	19,800	4325	9550	19,590
Heterophils (cell/μL)	5830 +/− 3206	1634	16,632	1828	5688	12,992
Eosinophils (cell/μL)	307 +/− 466	0	2166	8	159	1436
Basophils (cell/μL)	19 +/− 43	0	128	0	0	120
Lymphocytes (cell/μL)	3087 +/− 1563	930	7372	946	3192	6337
Monocytes (cell/μL)	446 +/− 375	52	1552	100	345	1465
H/L ratio	2.4 +/− 2.1	0.6	10.5	0.7	1.8	7.7

Note: RBC = red blood count, MCH = mean corpuscular haemoglobin, MCHC = mean corpuscular haemoglobin concentration, MCV = mean corpuscular volume, WBC = white blood count, H/L = heterophil-lymphocyte ratio, SD = standard deviation, Min = minimum value, Max = maximum value.

**Table 2:** Mean ± standard deviation, minimum and maximum values and quartiles and of blood chemistry variables of captive black-faced ibis (*Theristicus melanopis*) (N = 11).

Variable	Mean +/− SD	Min	Max	2.5%	50%	97.5%
Glucose (mg/dL)	209 +/− 84	79	302	81	254	298
Total proteins (g/dL)	4.4 +/− 1.8	0.6	6.9	1.2	4.1	6.9
Albumin (g/dL)	2.1 +/− 0.8	1.2	3.5	1.2	1.7	3.4
Globulin (d/dL)	3.1 +/− 1.3	1.7	6.1	1.8	2.7	5.7
Calcium (mg/dL)	8.8 +/− 1.5	6.3	10.6	6.4	9.1	10.6
Phosphorus (mg/dL)	9.9 +/− 5.3	3.2	20.9	3.8	8.9	20.2
BUN (mg/dL)	7.59 +/− 7.9	1.9	27.8	2.1	4.7	25
Creatinine (mg/dL)	0.5 +/− 0.3	0.0	0.8	0.05	0.5	0.8
ALP (IU/L)	402.8 +/− 179.4	195.8	715.5	199.1	353.6	708.4
AST (IU/L)	241 +/− 75	122	410	134	227	381
GGT (IU/L)	5.9 +/− 4.2	1.8	13.9	1.9	4.2	13.5
LDH (IU/L)	1485 +/− 819	612	3600	639	1477	3183
CPK (IU/L)	995 +/− 342	403	1423	453	1040	1418
A/G ratio	0.68 +/− 0.1	0.51	1.16	0.52	0.57	1.08

Note: BUN = blood urea nitrogen, ALP = alkaline phosphatase, AST = aspartate aminotransferase, GGT = gamma-glutamyl transpeptidase, LDH = lactate dehydrogenase, CP = creatine phosphokinase, A/G = albumin-globulin ratio, SD = standard deviation, Min = minimum value, Max = maximum value.

**Table 3:** Mean ± standard deviation of hematological variables of captive black-faced ibis (*Theristicus melanopis*) according to sampling location (N = 22).

Variable	La Serena (*n* = 3)	Santiago (*n* = 11)	Rancagua (*n* = 6)	Linares (*n* = 2)	*p* Value
RBC (10^6^ cells/μL)	2.97 +/− 0.04	3.07 +/− 0.49	3.08 +/− 0.54	2.68 +/− 0.50	0.73
Hematocrit (%)	50.3 +/− 1.5	49.1 +/− 4.7	52.3 +/− 2.8	46.5 +/− 9.1	0.49
Hemoglobin (g/dL)	17.3 +/− 0.6	16.5 +/− 2.7	17.4 +/− 1.6	15.5 +/− 3.7	0.71
MCH (pg/cell)	52.8 +/− 2.2	57.2 +/− 4.0	59.4 +/− 4.0	57.3 +/− 3.0	0.08
MCHC (g/dL)	34.3 +/− 0.2	32.9 +/− 3.3	33.2 +/− 1.3	33.0 +/− 1.4	0.69
MCV (fL)	169.3 +/− 3.0	175.0 +/− 19.5	172.6 +/− 22.8	173.0 +/− 1.4	0.88
WBC (cell/μL)	11,400 +/− 7660	9263 +/− 3022	7733 +/− 2787	15,500 +/− 5515	0.12
Heterophils (cell/μL)	8568 +/− 7289	5249 +/− 2054	4615 +/− 1720	8562 +/− 1609	0.18
Eosinophils (cell/μL)	114 +/− 76	425 +/− 601	62 +/− 42	678 +/− 138	0.25
Basophils (cell/μL)	32.0 +/− 55	30.1 +/− 52	0.0 +/− 0.0	0.0 +/− 0.0	0.48
Lymphocytes (cell/μL)	2016 +/− 410	3150 +/− 1282	2767 +/− 1554	5310 +/− 2916	0.11
Monocytes (cell/μL)	670 +/− 642	379 +/− 221	288 +/− 105	950 +/− 851	0.09
H/L ratio	4.8 +/− 4.9	1.9 +/− 1.2	2.0 +/− 1.1	1.8 +/− 0.6	0.79

Note: RBC = red blood count, MCH = mean corpuscular haemoglobin, MCHC = mean corpuscular haemoglobin concentration, MCV = mean corpuscular volume, WBC = white blood count, H/L = heterophil-lymphocyte ratio.

**Table 4:** Mean ± standard deviation of blood biochemistry variables of captive black-faced ibis (*Theristicus melanopis*) according to sampling location (N = 11).

Variable	Santiago (*n* = 3)	Rancagua (*n* = 6)	Linares (*n* = 2)	*p* Value
Glucose (mg/dL)	159 +/− 105	208 +/− 75	283 +/− 26	0.29
Total proteins (g/dL)	4.6 +/− 1.9	3.9 +/− 1.9	5.3 +/− 2.3	0.68
Albumin (g/dL)	1.6 +/− 0.6	2.2 +/− 0.9	2.4 +/− 0.9	0.69
Globulin (d/dL)	2.9 +/− 1.4	3.3 +/− 1.5	2.9 +/− 1.3	0.92
Calcium (mg/dL)	8.9 +/− 2.3	9.0 +/− 1.4	7.9 +/− 0.4	0.69
Phosphorus (mg/dL)	10.0 +/− 7.5	10.0 +/− 5.5	9.6 +/− 4.3	0.99
BUN (mg/dL)	2.7 +/− 0.6	6.6 +/− 5.2	17.8 +/− 14.1	0.08
Creatinine (mg/dL)	0.4 +/− 0.3	0.5 +/− 0.3	0.4 +/− 0.0	0.76
ALP (IU/L)	413.1 +/− 270.0	404.4 +/− 151.3	382.4 +/− 245.4	0.98
AST (IU/L)	206 +/− 75	257 +/− 87	243 +/− 34	0.66
GGT (IU/L)	5.0 +/− 1.5	6.9 +/− 5.6	4.5 +/− 0.5	0.74
LDH (IU/L)	1952 +/− 1484	1178 +/− 421	1705 +/− 323	0.41
CPK (IU/L)	777 +/− 231	977 +/− 353	1379 +/− 36	0.15
A/G ratio	0.59 +/− 0.10	0.68 +/− 0.24	0.83 +/− 0.02	0.16

Note: BUN = blood urea nitrogen, ALP = alkaline phosphatase, AST = aspartate aminotransferase, GGT = gamma-glutamyl transpeptidase, LDH = lactate dehydrogenase, CPK = creatine phosphokinase, A/G = albumin-globulin ratio.

**Table 5:** Mean ± standard deviation of hematological variables of captive black-faced ibis (*Theristicus melanopis*) according to sex (N = 22).

Variable	Male (*n* = 12)	Female (*n* = 10)	*p* Value
RBC (10^6^ cells/μL)	3.16 +/− 0.53	2.86 +/− 0.31	0.11
Hematocrit (%)	49.5 +/− 4.9	50.3 +/− 4.3	0.68
Hemoglobin (g/dL)	17.3 +/− 2.7	16.1 +/− 1.5	0.22
MCH (pg/cell)	56.8 +/− 4.4	57.9 +/− 3.9	0.55
MCHC (g/dL)	33.9 +/− 2.9	32.5 +/− 1.7	0.19
MCV (fL)	171.3 +/− 20.4	176.0 +/− 14.6	0.55
WBC (cell/μL)	9742 +/− 3867	9660 +/− 4884	0.97
Heterophils (cell/μL)	5913 +/− 3715	5730 +/− 2662	0.89
Eosinophils (cell/μL)	354 +/− 595	259 +/− 250	0.61
Basophils (cell/μL)	25.0 +/− 45.5	12.8 +/− 40.5	0.52
Lymphocytes (cell/μL)	3025 +/− 1108	3161 +/− 2047	0.85
Monocytes (cell/μL)	412 +/− 320	486 +/− 447	0.66
H/L ratio	2.4 +/− 2.6	2.3 +/− 1.3	0.86

Note: RBC = red blood count, MCH = mean corpuscular haemoglobin, MCHC = mean corpuscular haemoglobin concentration, MCV = mean corpuscular volume, WBC = white blood count, H/L = heterophil-lymphocyte ratio.

**Table 6:** Mean ± standard deviation of blood biochemistry variables of captive black-faced ibis (*Theristicus melanopis*) according to sex (N = 11).

Variable	Male (*n* = 3)	Female (*n* = 8)	*p*-Value
Glucose (mg/dL)	273 +/− 17	184 +/− 87	0.12
Total proteins (g/dL)	3.6 +/− 2.8	4.6 +/− 1.5	0.43
Albumin (g/dL)	2.4 +/− 1.1	1.9 +/− 0.7	0.46
Globulin (d/dL)	4.2 +/− 1.7	2.7 +/− 0.9	0.08
Calcium (mg/dL)	9.5 +/− 1.1	8.5 +/− 1.6	0.38
Phosphorus (mg/dL)	11.0 +/− 9.0	9.5 +/− 4.1	0.71
BUN (mg/dL)	9.3 +/− 6.8	6.9 +/− 8.6	0.68
Creatinine (mg/dL)	0.7 +/− 0.1	0.4 +/− 0.3	0.13
ALP (IU/L)	549.2 +/− 263.7	347.9 +/− 116.1	0.09
AST (IU/L)	228 +/− 29	246 +/− 87	0.76
GGT (IU/L)	3.6 +/− 2.8	6.8 +/− 4.4	0.28
LDH (IU/L)	695 +/− 374	1781 +/− 868	0.10
CPK (IU/L)	1117 +/− 393	672 +/− 360	0.12
A/G ratio	0.55 /−0.02	0.74 +/− 0.21	0.17

Note: BUN = blood urea nitrogen, ALP = alkaline phosphatase, AST = aspartate aminotransferase, GGT = gamma-glutamyl transpeptidase, LDH = lactate dehydrogenase, CPK = creatine phosphokinase, A/G = albumin-globulin ratio.

## References

[1] Vuilleumier F. (1985). Forest birds of patagonia: Ecological geography, speciation, endemism, and faunal history. Ornithol. Monogr..

[2] BirdLife International (2017). *Theristicus* *melanopis*. IUCN Red List of Threatened Species.

[3] Azpiroz A.B., Isacch J.P., Dias R.A., Di Giacomo A.S., Fontana C.S., Palarea C.M. (2012). Ecology and conservation of grassland birds in southeastern South America: A review. J. Field Ornithol..

[4] Raimilla V., Rau J.R., Niklitschek E.J. (2015). Use of exotic conifers as nesting sites by black-faced ibis (*Theristicus melanopis melanopis*) in an urban area of southern Chile. Stud. Neotrop. Fauna Environ..

[5] Gantz A., Yañez M. (2016). Breeding biology of the black-faced ibis (*Theristicus melanopis*) in Southern Chile. Waterbirds.

[6] SAG (2015). Servicio Agricola y Ganadero Ley de Caza.

[7] Del Hoyo J., Elliott A., Sargatal J., del Hoyo J., Elliott A., Sargatal J. (1992). Family Threskiornithidae (ibises and spoonbills). Handbook of the Birds of the World, Vol. 1, Ostriches to Ducks.

[8] Gantz A., Schlatter R. (1995). La dieta de la bandurria (*Theristicus caudatus melanopis* Gmelin, 1789) en praderas agrícolas del sur de Chile. Medio Ambiente.

[9] Cornelius C., Cofre H., Marquet P.A. (2000). Effects of habitat ƒragmentation on bird species in a relict temperate forest in semiarid Chile. Conserv. Biol..

[10] Hernandez C.L., Oster S.C., Newbrey J.L. (2018). Retrospective study of raptors treated at the southeastern raptor center in Auburn, Alabama. J. Raptor Res..

[11] Fix A.S., Barrows S.Z. (1990). Raptors rehabilitated in Iowa during 1986 and 1987: A retrospective study. J. Wildl. Dis..

[12] Doussang D., Palma C., Moreno L., Zambrano B., Pavéz E., Cerda F., González-Acuña D. (2018). Hematological and Biochemical Parameters of Captive Andean Condors. J. Raptor Res..

[13] Molina-López R.A., Casal J., Darwich L. (2015). Prognostic indicators associated with early mortality of wild raptors admitted to a wildlife rehabilitation centre in Spain. Vet. Q..

[14] Parsons N.J., Vanstreels R.E.T., Schaefer A.M. (2018). Prognostic indicators of rehabilitation outcomes for adult african penguins (*Spheniscus demersus*). J. Wildl. Dis..

[15] Stanclova G., Schwendenwein I., Merkel O., Kenner L., Dittami J., Fritz J., Scope A. (2017). The effects of migratory flight on hematologic parameters in northern bald ibises (*Geronticus eremita*). J. Zoo Wildl. Med..

[16] Artacho P., Soto-Gamboa M., Verdugo C., Nespolo R.F. (2007). Using haematological parameters to infer the health and nutritional status of an endangered black-necked swan population. Comp. Biochem. Physiol. Part A Mol. Integr. Physiol..

[17] Minias P. (2015). The use of haemoglobin concentrations to assess physiological condition in birds: A review. Conserv. Physiol..

[18] Kilkenny C., Browne W.J., Cuthill I.C., Emerson M., Altman D.G. (2010). Improving Bioscience Research Reporting: The ARRIVE Guidelines for Reporting Animal Research. Plos Biol..

[19] Horowitz G., Burtis C., Bruns D. (2015). Establishment and use of reference values. Tietz Fundamentals of Clinical Chemistry and Molecular Diagnostics.

[20] R Development Core Team (2014). R: A Language and Environment for Statistical Computing.

[21] Friedrichs K.R., Harr K.E., Freeman K.P., Szladovits B., Walton R.M., Barnhart K.F., Blanco-Chavez J. (2012). American Society for Veterinary Clinical Pathology ASVCP reference interval guidelines: Determination of de novo reference intervals in veterinary species and other related topics. Vet. Clin. Pathol..

[22] Dutton C.J., Allchurch A.F., Cooper J.E. (2002). Comparison of hematologic and biochemical reference ranges between captive populations of northern bald ibises (*Geronticus eremita*). J. Wildl. Dis..

[23] Coke R.L., West G.D., Hoover J.P. (2004). Hematology and plasma ßiochemistry of captive Puna ibis (*Plegadis ridgewayi*). J. Wildl. Dis..

[24] Puerta M.L., Pulido R.M., Huecas V., Abelenda M. (1989). Hematology and blood chemistry of chicks of white and black storks (*Ciconia ciconia* and *Ciconia nigra*). Comp. Biochem. Physiol. Part A Physiol..

[25] Alonso J.C., Huecas V., Alonso J.A., Abelenda M., Muñoz-Pulido R., Puerta M.L. (1991). Hematology and blood chemistry of adult white storks (*Ciconia ciconia*). Comp. Biochem. Physiol. Part A Physiol..

[26] Han J., Jang H., Na K. (2016). Hematologic and serum biochemical reference intervals of the oriental white stork (*Ciconia boyciana*) and the application of an automatic hematologic analyzer. J. Vet. Sci..

[27] Villegas A., Guzmán J.M.S., Corbacho C., Corbacho P., Vargas J.M. (2004). Blood values of bald ibis (*Geronticus eremita*) in captivity: Comparative ranges and variability with age, sex and physical condition. J. Ornithol..

[28] Celdrán J., Polo F.J., Peinado V.I., Viscor G., Palomeque J. (1994). Haematology of captive herons, egrets, spoonbill, ibis and gallinule. Comp. Biochem. Physiol. Part A Physiol..

[29] BirdLife International (2016). *Geronticus* *calvus*. IUCN Red List of Threatened Species.

[30] BirdLife International (2016). *Plegadis* *falcinellus*. IUCN Red List of Threatened Species.

[31] BirdLife International (2016). *Plegadis* *ridgway*. IUCN Red List of Threatened Species.

[32] Norambuena M.C., Bozinovic F. (2009). Effect of malnutrition on iron homeostasis in black-necked swans (*Cygnus melanocoryphus*). J. Zoo Wildl. Med..

[33] Mete A., Hendriks H.G., Klaren P.H.M., Dorrestein G.M., van Dijk J.E., Marx J.J.M. (2003). Iron metabolism in mynah birds (*Gracula religiosa*) resembles human hereditary haemochromatosis. Avian Pathol..

[34] Fassbinder-Orth C.A., Karasov W.H. (2006). Effects of feed restriction and realimentation on digestive and immune function in the Leghorn chick. Poult. Sci..

[35] Graczyk T.K., Cranfield M.R., Bicknese E.J. (1995). Evaluation of serum chemistry values associated with avian malaria infections in African black-footed penguins (*Spheniscus demersus*). Parasitol. Res..

[36] Moberg G.P., Mench J.A. (2000). The Biology of Animal Stress: Basic Principles and Implication for Animal Welfare.

[37] Vleck C.M., Vertalino N., Vleck D., Bucher T.L. (2000). Stress, corticosterone, and heterophil to lymphocyte ratios in free-living Adélie penguins. Condor.

